# A Sentimental Sketch

**Published:** 1888-03

**Authors:** 


### A SENTIMENTAL SKETCH.

On Brooklyn Heights the scene is laid,—
The hour is twilight—actors, two ;
A dudish youth, a frizzled maid,
In tennis badge of tender blue.

"These English sparrows,—pshaw!" said she,
"They so defile our brownstone homes
I wish them gone." "Well, yes," said he,
"But then they're pref'rable to worms."

Thus, in good nature, held debate
Of worms and sparrows, which to have
Were worse or better,—John and Kate,—
When Tom came in, a wheelman brave.

Left to decide between the two—
"I know, said Tom, "that worms are bad,
They make a fellow feel so blue,
But *sparrows* I have never had." DE T.

